# Application of Jejunal Nutrient Tube in Congenital Duodenal Obstruction in Children

**Published:** 2018-04

**Authors:** Junhua CAO, Ying CAI, Zhenfang QIN, Xiaobing XU, Xu LIU, Peng LU, Huaxin ZOU, Yiyu YIN

**Affiliations:** 1. Dept. of Emergency Medicine, Xuzhou Children’s Hospital, Xuzhou 221006, P.R. China; 2. Dept. of Nursing, Xuzhou Children’s Hospital, Xuzhou 221006, P.R. China; 3. Dept. of General Surgery, Xuzhou Children’s Hospital, Xuzhou 221006, P.R. China

## Dear Editor-in-Chief

Congenital duodenal obstruction (CDO) in children is a kind of common upper gastrointestinal malformation with various pathological types, caused by the abnormal embryonic development, including duodenal stenosis and atresia, annular pancreas. CDO in children is often manifested as abdominal distension, frequent and severe bilious vomiting; such children often need to receive the operation to establish the continuous digestive tract, and to be fasted after operation to reduce the risk of anastomotic fistula. Therefore, the postoperative nutritional support appears particularly important. The jejunal nutrient tube has been increasingly used in child and adult critical patients in recent years (1–2), so as to provide early appropriate enteral nutrition (EN).

The present study investigated the role of jejunal nutrient tube in postoperative enteral nutrition support for child patients with CDO.

The clinical data of 17 child patients with CDO (observation group) receiving surgical treatment and intraoperative placement of jejunal nutrient tube for early enteral nutrition from January 2013 to June 2017 were analyzed retrospectively. Fifteen child patients with CDO (control group) receiving laparoscopic surgery but without undergoing the enteral nutrition via jejunal nutrient tube in our hospital were selected. The perioperative period, postoperative complications and changes in some nutritional indexes were compared between the two groups. Informed consent was taken from the participants and the study was approved by the hospital authorities.

All of 17 child patients (Table 1–3) in observation group were treated with laparoscopic duodenal diamond-shaped anastomosis and placed with the jejunal nutrient tube successfully. The operation time was 60–105 min with an average of 80.9 min. There was no postoperative anastomotic fistula and intestinal perforation. The average recovery time of intestinal function after operation was 14.9 h, which was shorter than that in control group, and the difference was statistically significant (*P*<0.05). The increase ranges of serum total protein, albumin and body weight after operation in observation group were significantly larger than those in control group (*P*<0.001).

**Table 1:** Comparisons of general data between observation group and control group

	***Case (n)***	***Age at admission (d)***	***Weight (g)***	***Gender***		***Premature infant***		***Malformation***	
				***Male***	***Female***	***Yes***	***No***	***Yes***	***No***
Observation group	17	7.11±4.24	2.88±0.48	10	7	8	9	3	14
Control group	15	6.33±3.94	3.02±0.35	9	6	7	8	3	12
*t* (χ^2^)		0.540	−0.910	(0.005)		(0.125)		(0.029)	
*P*		0.593	0.370	0.946		0.723		0.965	

**Table 2:** Perioperative period and postoperative complications of child patients in observation group and control group

***Group***	***n***	***Operation time (min)***	***Recovery time of intestinal function (h)***	***Complication***		
				***Vomiting (n)***	***Diarrhea (n)***	***abdominal distension (n)***
Observation group	17	80.9±9.6	14.9±5.1	2*	2	1
Control group	15	72.7±9.8	25.3±5.8	1	1	1
*t* (χ^2^)		*t*=2.398	*t*=−5.371	χ^2^=0.376		
*P*		0.023	0.000	0.539		

* 1 case of vomiting due to intolerance to jejunal nutrient tube

**Table 3:** Comparisons of some nutritional indexes at 7 d after operation and at 1 d before operation between observation group and control group

***Group***	***n***	***Increase range of total protein (g/L)***	***Increase range of albumin (g/L)***	***Increase range of hemoglobin (g/L)***	***Increase range of weight (kg)***
Observation group	16*	5.09±1.26	3.33±0.85	5.93±1.61	0.16±0.06
Control group	15	3.81±0.79	2.33±0.69	5.10±1.22	0.10±0.04
*t*		3.406	3.561	1.596	3.400
*P*		0.02	0.01	0.121	0.002

* 1 case of intolerance to jejunal nutrient tube eliminated from observation group

EN refers to the injection of nutrients required for the body to maintain the basic functions or growth and development into the patient’s intestine through different ways, replacing the gastric digestive function, which can provide necessary nutritional support for child patients with gastric digestive dysfunction (3).

With the understanding of the EN in children and the update of catheter material and inserting technique, EN via jejunal nutrient tube in children has been gradually recognized by Chinese Society of Parenteral and Enteral Nutrition (4). In this study, the prime reason for the observation of significantly shorter recovery time of intestinal function in observation group might be that the early postoperative EN via jejunal nutrient tube in observation group that might have effectively promoted the secretion of digestive juice and intestinal peristalsis. So, the postoperative defecation time in observation group was quite early in comparison to control group and the results were in sync with earlier observations (5). Further, the improvement of nutritional indexes at 7 d after operation was inspiring: The increase ranges of serum total protein, albumin and body weight in observation group were significantly higher than those in control group. Therefore, intraoperative placement of jejunal nutrient tube for early EN support for child patients with CDO is an important means of postoperative nutritional support that not only promotes the recovery of intestinal function, but also improves the postoperative nutritional status of child patients.
